# Recognition of the Effect of Indirect Revascularization for Moyamoya Disease: The Balance Between the Stage Progression and Neoangiogenesis

**DOI:** 10.3389/fneur.2022.861187

**Published:** 2022-05-06

**Authors:** Xiang-Yang Bao, Qian-Nan Wang, Xiao-Peng Wang, Ri-Miao Yang, Zheng-Xing Zou, Qian Zhang, De-Sheng Li, Lian Duan

**Affiliations:** ^1^Department of Neurosurgery, Chinese PLA General Hospital (Former Department of Neurosurgery, The Fifth Medical Center of Chinese PLA General Hospital), Beijing, China; ^2^Department of Neurosurgery, Chinese PLA General Hospital (Former Department of Neurosurgery, The Eighth Medical Center of Chinese PLA General Hospital), Beijing, China; ^3^Department of Neurosurgery, 307th Hospital of People's Liberation Army, 307 Clinical College, Anhui Medical University, Hefei, China

**Keywords:** moyamoya, neoangiogenesis, encephaloduroarteriosynangiosis, long-term, Matsushima grade

## Abstract

**Objective:**

To explore the long-term progression of neoangiogenesis after indirect revascularization for moyamoya disease (MMD).

**Methods:**

We enrolled patients who were diagnosed with MMD and treated by encephaloduroarteriosynangiosis (EDAS) surgery at our center from December 2002 through September 2009. A comparative study between short-term (6–12 months) and long-term (duration ≥ 8 years) follow-up angiographies was performed. The development of collateral circulation through EDAS was graded according to the system described by the Matsushima grade system.

**Results:**

A total of 78 patients who received indirect EDAS were enrolled in the study. The mean age at the first operation was 26.9 ± 15.0 years. The Matsushima grades of the same hemisphere were higher at the long-term follow-up compared with the short-term follow-up. Importantly, no attenuation was observed in any hemisphere during the long-term follow-up. In total, 51 hemispheres (32.7%) and 26 hemispheres (16.6%) had progression during the short-term and the long-term follow-up, respectively. The ipsilateral Suzuki stage showed a significant negative correlation with progression pace. Furthermore, higher Suzuki stages were significantly correlated with the postsurgical Matsushima grade at both time points. A total of nine strokes (11.5%) occurred in 78 patients was reported at the long-term follow-up. The annual incidence rate of recurrent strokes was higher for the stage progression group than for the stable group.

**Conclusion:**

For patients with MMD, postsurgical neoangiogenesis after indirect bypass continuously improved with time. The short-term progression of the internal carotid artery (ICA) might be attributed to cerebral revascularization, while the long-term progression should be attributed to the natural progression of the disease.

## Introduction

Moyamoya disease (MMD) is a rare, chronic, and progressive cerebrovascular disorder that is characterized by stenosis and occlusion of the distal carotid, proximal middle, and anterior cerebral arteries and is accompanied by the development of small collateral vessel networks (1, 2). Currently, surgical revascularization is considered the standard treatment for MMD that could prevent strokes by augmenting blood flow to the affected cerebral hemispheres. The most used surgical modalities include direct bypass, indirect bypass, and combined bypass. Indirect revascularization, or indirect bypass surgery, is an essential component of surgical treatment for MMD. This procedure improves cerebral perfusion by attaching pedicled, vascularized grafts to the cortical surface and facilitating ingrowth of neoangiogenesis (3, 4).

Indirect encephaloduroarteriosynangiosis (EDAS) has been a widely established treatment strategy for patients with MMD because of its excellent postoperative results, leading to extensive collateral formation and minimal complications (5–9). However, the progression of neoangiogenesis after surgery has not yet been investigated. How long it takes for neovascularization to fully develop and whether the ingrowth continues in the long-term remain unknown. A recent study found that after indirect bypass surgery, neoangiogenesis was very similar at short-term (3–6 months) and long-term (1 year) follow-ups, indicating the most important period for extension of new vessels across the cortical surface was within 6 months of surgery (10). Conversely, another study found the proportion of cases with good neoangiogenesis after EDAS was higher when the patients underwent follow-up for more than 24 months, indicating a general trend of collateral grade improvement upon an increase in follow-up length (9). Therefore, we conducted the current retrospective study comparing neoangiogenesis at short-term (7.4 months) follow-up and long-term (12.5 years) follow-up after indirect bypass surgery, hoping to identify the proceed of the ICA after revascularization surgery.

## Methods

### Patient Selection

We enrolled patients who were diagnosed with MMD and treated at the Department of Neurosurgery in the Fifth Medical Center of Chinese PLA General Hospital, Beijing, China from December 2002 through September 2009. The inclusion criteria were as follows: (1) diagnosis of definite or probable MMD *via* digital subtraction angiography (DSA) (11); (2) patients who underwent EDAS by a single surgeon (Duan Lian) for the treatment of MMD; and (3) patients who received at least two postoperative DSAs after surgical revascularization, a short-term (6–9 months) and a long-term (duration ≥8 years after surgical revascularization up to the latest follow-up angiography). A total of 78 patients were included in this study. Many of the patients included in this study received several postoperative DSAs between the first and the last follow-up angiography. Surgical procedures were described in our previous reports (12, 13). Bilateral surgery is generally required for patients with definitive MMD. In bilateral MMD, we first revascularized the clinically more symptomatic side or more serious hemodynamic impairment. If no lateralizing signs or symptoms were present, we preferred to revascularize the dominant side first. The second side was usually revascularized 3 months after the first surgery, as tolerated by the patient. In patients who underwent bilateral surgery, the duration of follow-up began from the time of the last operation. The study was approved by the Research Ethics Board at the Fifth Medical Center of Chinese PLA General Hospital and all the subjects provided written informed consent to participate in the study. All the procedures performed in this study involving human participants were in accordance with the Declaration of Helsinki (1964). The clinical trial was registered at the ClinicalTrials.gov identifier (NCT number): NCT03627975.

### Clinical Data Collection

Information on patient sex, age at admission, family history of MMD, initial symptomatic presentation at diagnosis, and radiographic presentations at admission was recorded. Onset type of MMD was defined as hemorrhagic (intracranial hemorrhage, subarachnoid hemorrhage, or intraventricular hemorrhage), ischemic (cerebral infarction or TIA), or atypical (seizure, headache, or asymptomatic) based on the first presenting symptoms.

### Evaluation of Vasculature Characteristics

Digital subtraction angiography (DSA), including injection of both internal carotid arteries and both vertebral arteries, as well as assessment through the late venous phase to evaluate the collateral flow from all possible sources, was required for all recruited patients preoperatively and on follow-up. All angiographic characteristics were evaluated by two experienced neurosurgeons who were blinded to genotypes, including Suzuki stages (2), the involvement of posterior cerebral artery (PCA), collateral circulation, superficial temporal artery (STA) collateral, middle meningeal artery (MMA) collateral, and occipital artery (OA) collateral. Discrepancies in the radiologic findings were discussed before a final decision was made.

The formation of collaterals by major intracranial and extracranial arteries was evaluated by their presence or absence, including the anterior cerebral artery (ACA), ophthalmic artery (OphA), posterior cerebral artery (PCA), posterior choroidal artery (PchA), and posterior communicating artery (PcoA). Any existing PCA-, PchA-, or PcoA-generated collateral vessels were defined as posterior–anterior collaterals. Any MMA-, STA-, or OcciA-generated collateral vessels were defined as spontaneous extracranial–intracranial (EC–IC) collaterals.

### Evaluation of Postsurgical Neoangiogenesis and the Stage Progression

**Figure 1:**
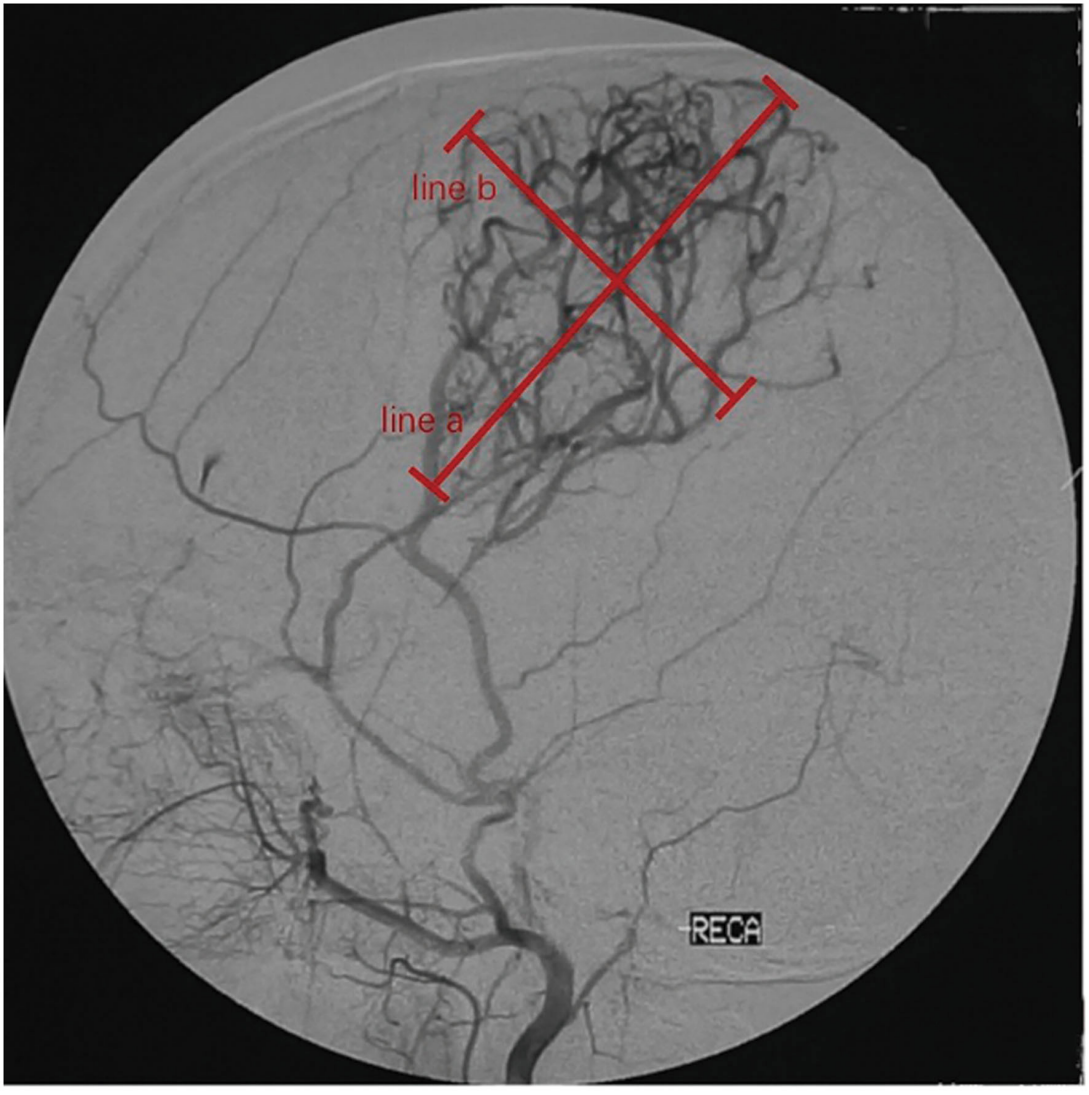
Quantitative measurements on follow-up digital subtraction angiography (DSA). Measurements were taken on the lateral view. Width (line a) was measured as the longest horizontal distance of the area covered by neoangiogenesis. Height (line b) was measured as the longest vertical distance of the area covered by neoangiogenesis.

To assess the efficacy of neoangiogenesis and guide subsequent treatment management, we requested that each patient undergo follow-up cerebral arteriography after surgery at 6 months, 1 year, and each following year. The development of collateral circulation through EDAS was graded according to the system described by Matsushima et al. (14): grade 3, in which the area supplied by EDAS covered more than two-thirds of the MCA distribution; grade 2, in which one-third to two-thirds of the MCA distribution was covered; grade 1, in which only one-third of the MCA distribution was covered through EDAS; grade 0, no collateral circulation was observed. The patients were divided into a good postoperative collateral circulation group (grade 2 or grade 3) and a poor postoperative collateral circulation group (grade 0 or grade 1). To evaluate postsurgical neoangiogenesis more accurately, the longest horizontal distance (width) and vertical distance (height) of the area covered by neoangiogenesis were measured as in a previous study (10) (Figure 1). Then, the progression of lesions from lower to higher Suzuki stages was divided into two levels: mild progression, progressing from the initial Suzuki stage into the next stage, which we define it as the stable group; severe progression, progressing into the higher Suzuki stage by striding over 2 or more stages, we define it as the progression group. Meanwhile, we evaluated long-term clinical outcomes by evaluating the occurrence of recurrent strokes (cerebral infarction or hemorrhage).

### Statistical Analysis

All the clinical characteristic data are presented as mean ± SD for continuous variables and *n* (%) for categorical variables. The categorical variables were analyzed using the chi-square (χ^2^) test, the continuous variables were compared using the paired *t*-test or Wilcoxon signed-rank test, multiple linear regression, and Spearman's correlation analysis was also conducted. The Kaplan–Meier survival analysis was used to estimate stroke risk. The data were considered statistically significant when the *p*-value was <0.05. All the statistical analyses were conducted with IBM SPSS statistical software version 22 for Windows (IBM Corp., Armonk, NY, USA).

### Data Availability Statement

With the permission of the corresponding authors and their institutions, combined with the relevant documents, all the data used for analysis will be shared after ethics approval if requested by other investigators for reasonable purposes of replicating procedures and results.

## Results

### Demographic Data

A total of 78 patients who received indirect EDAS were enrolled in the study. Their clinical characteristics are summarized in Table 1. The mean ± SD age at the first operation was 26.9 ± 15.0 years (range 2–55 years), and there were 42 female and 36 male patients (women/men ratio was 1.17:1.00). In total, 34 patients were diagnosed with either hypertension, diabetes mellitus (DM), or hyperlipidemia. Among the 78 patients with MMD, 56 (71.8%) exhibited ischemic symptoms, 14 (18.0%) exhibited hemorrhagic symptoms, and eight (10.2%) exhibited atypical symptoms as the initial symptoms.

**Table 1:** Baseline characteristics of patients.

**Patient characteristics**	**Number of patients/ hemispheres (*n* = 78/156)**
Mean age ± SD, y	26.9 ± 15.0
Female	42 (53.8%)
Unilateral Lesions	14 (17.9%)
PCI	38 (48.7%)
Stroke risk factors	
Hypertension	15 (19.2%)
Diabetes mellitus	6 (7.7%)
Hyperlipidemia	13 (16.7%)
Smoking or drinking	11 (14.1%)
Suzuki Angiographic Stage	
1	2 (1.3%)
2	4 (2.6%)
3	22 (14.1%)
4	34 (21.8%)
5	58 (37.2%)
6	36 (23.1%)
Posterior communicating artery collaterals	73 (46.8%)
External carotid artery collaterals	49 (31.4%)
Ophthalmic artery collaterals	27 (17.3%)

Of the 78 patients who received EDAS procedures, 62 were bilateral. Of the untreated 16 hemispheres, 14 were unilateral cases with unaffected hemispheres and two patients refused contralateral surgery. Of the 14 unilateral cases, five progressed to bilateral lesion during follow-up and eventually received EDAS procedures. A total of 145 EDAS procedures were performed.

### Comparison of Neoangiogenesis at Short-Term and Long-Term Follow-Up

In 156 hemispheres, the short-term follow-up angiographies were conducted at a mean of 7.4 ± 2.6 months (range 5.6–13.4 months) after surgery. The long-term follow-up angiographies were conducted at a mean of 12.5 ± 3.7 years (range 8.1–17.8 years) after surgery. At the short-term angiography, 29 (20.7%) hemispheres achieved the Matsushima grade 3, 35 (25.0%) achieved the Matsushima grade 2, 46 (32.9%) achieved Matsushima grade 1, and 30 (21.4%) achieved Matsushima grade 0. At the long-term follow-up, 45 (31.0%) hemispheres achieved Matsushima grade 3, 49 (33.8%) achieved Matsushima grade 2, 27 (18.6%) achieved Matsushima grade 1, and 24 (16.6%) were Matsushima grade 0. The Matsushima grades of the same hemisphere were higher at the long-term follow-up compared with the short-term follow-up (*P* = 0.010, Table 2). Importantly, no attenuation was observed in any hemisphere during the long-term follow-up, demonstrating that postsurgical neoangiogenesis improved over time.

**Table 2:** Comparison of neoangiogenesis at short-term and long-term follow-up.

	**Short-term**	**Long-term**	***p***
Matsushima grade			0.010
Grade 0	30	24	
Grade 1	46	27	
Grade 2	35	49	
Grade 3	29	45	

Quantitative measurements were also compared between different follow-up intervals. The width of neoangiogenesis at the long-term follow-up was higher than that at the short-term follow-up (102.4 ± 47.2 vs. 87.4 ± 38.1, *P* = 0.028). The same trends were observed for height (94.3 ± 29.1 vs. 71.3 ± 24.4, *P* = 0.036).

At the long-term follow-up, among the 145 hemispheres that underwent EDAS surgery, 94 (64.8%) hemispheres had good neoangiogenesis after surgery and 51 (35.2%) had poor development. Univariate analysis showed that younger age and existence of spontaneous EC–IC collaterals were significantly related to good neoangiogenesis (*p* = 0.012 and *p* = 0.032, respectively), whereas hemorrhagic onset and diagnosed hypertension were related to poor neoangiogenesis (*p*= 0.009 and *p* = 0.038, respectively). To further rule out the effects of various factors, a multivariate analysis was conducted with the aforementioned significant factors. Hemorrhagic onset (*p* = 0.011) was recognized as an independent predictor for neoangiogenesis after EDAS surgery.

### Stage Progression and Clinical Outcome at Follow-Up

**Figure 2:**
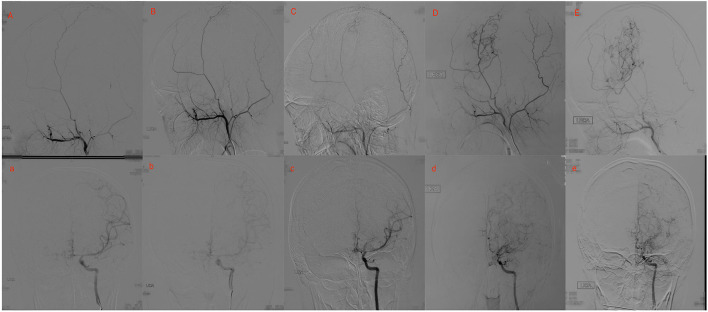
Twenty yrs, female. **(A)** a: Preoperative angiography showed mild stenosis at the proximal to the left middle cerebral artery (MCA) and anterior cerebral artery (ACA; Suzuki gradeI), and well-developed left superficial temporal artery (STA). **(B)** b: Postoperative angiography 2.5 years after EDAS showed the stenosis was almost unchanged (Suzuki 1 gradeI), and no collateral circulation through EDAS was observed. **(C)** c: Postoperative angiography 3.5 years after EDAS showed remarkable progress in the stenosis of the proximal of MCA and ACA, and collateral circulation through EDAS began to develop. **(D)** d: Postoperative angiography 8 years after EDAS showed occlusion of left MCA and ACA with intensification of abnormal vascular networks (Suzuki grade III), and more than one-third of the MCA distribution was covered by collateral circulation through EDAS. **(E)** e: Postoperative angiography 10.1 years after EDAS showed occlusion of left MCA and ACA with minimization of abnormal vascular networks (Suzuki grade IV), and more than two-thirds of the MCA distribution was covered by collateral circulation through EDAS.

**Figure 3:**
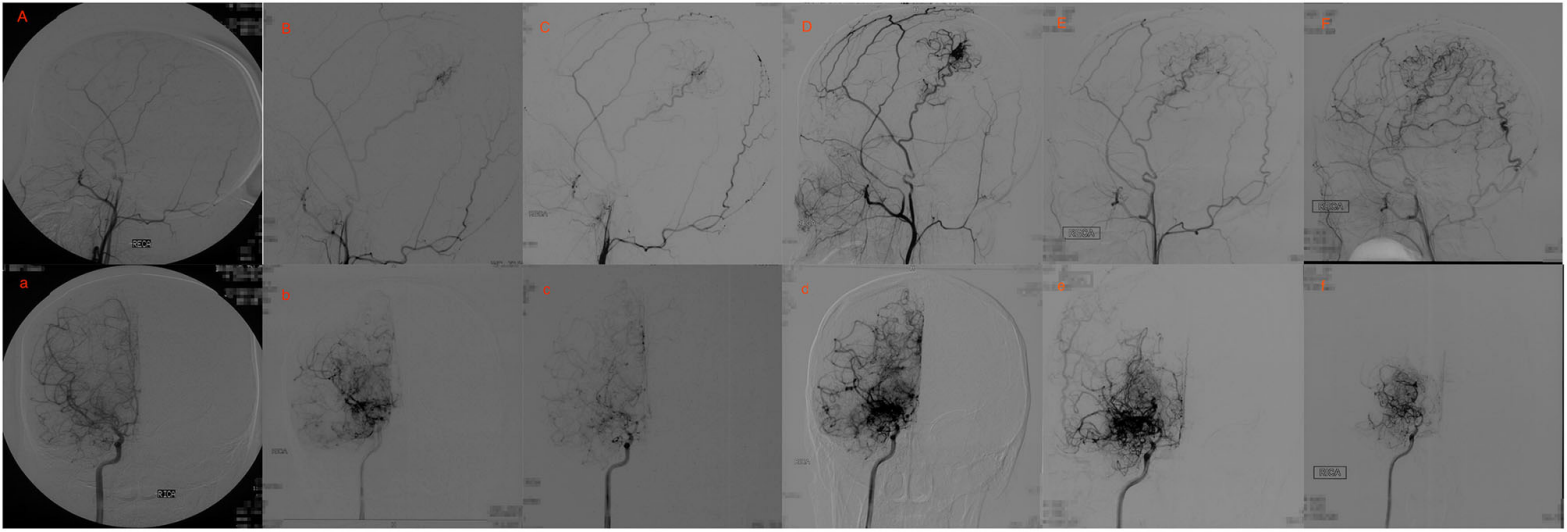
Eighteen yrs, female. **(A)** a: Preoperative angiography showed stenosis at the proximal of right MCA and ACA (Suzuki gradeI), and well-developed right STA. **(B,C)** b,c: Postoperative angiography 0.7 and 2 years after EDAS showed almost occlusion of the right MCA and ACA with the initiation of abnormal vascular networks (Suzuki gradeII), and collateral circulation through EDAS began to develop. **(D,E)** d,e: Postoperative angiography 3 and 8 years after EDAS showed occlusion of right MCA and ACA with intensification of abnormal vascular networks (Suzuki grade III), and one third to two-thirds of the MCA distribution was covered by collateral circulation through EDAS. **(F)** f: Postoperative angiography 11 years after EDAS showed occlusion of left MCA and ACA with reduced abnormal vascular networks (Suzuki grade IV), and more than two-thirds of the MCA distribution was covered by collateral circulation through EDAS.

During the short-term follow-up, stage progression with different levels was observed in 50 operative hemispheres and only one non-operative hemisphere (35.7 vs. 6.3%, respectively; *p* = 0.017). At short-term follow-ups, 32 (20.5%) hemispheres had mild progression and 19 (12.2%) had severe progression. At long-term follow-ups, 16 (10.2%) hemispheres had mild progression and 10 (6.4%) hemispheres had severe progression. The stage progression at short-term follow-ups showed more rapid progress compared with that at long-term follow-ups. The ipsilateral Suzuki stage showed a significant negative correlation with progression pace, where the higher Suzuki stage showed slower progression (OR = 0.612, *P* = 0.004). To assess whether neovascularization after EDAS correlates with postoperative progressive occlusion of ICA, Spearman correlation analysis between Matsushima level and increase of Suzuki stage was performed. We found increased Suzuki stage was significant correlated with the postsurgical Matsushima grade at both time points (short-term: *P* = 0.042; long-term: *P* = 0.017). Two typical cases are shown in Figures 2, 3.

**Figure 4:**
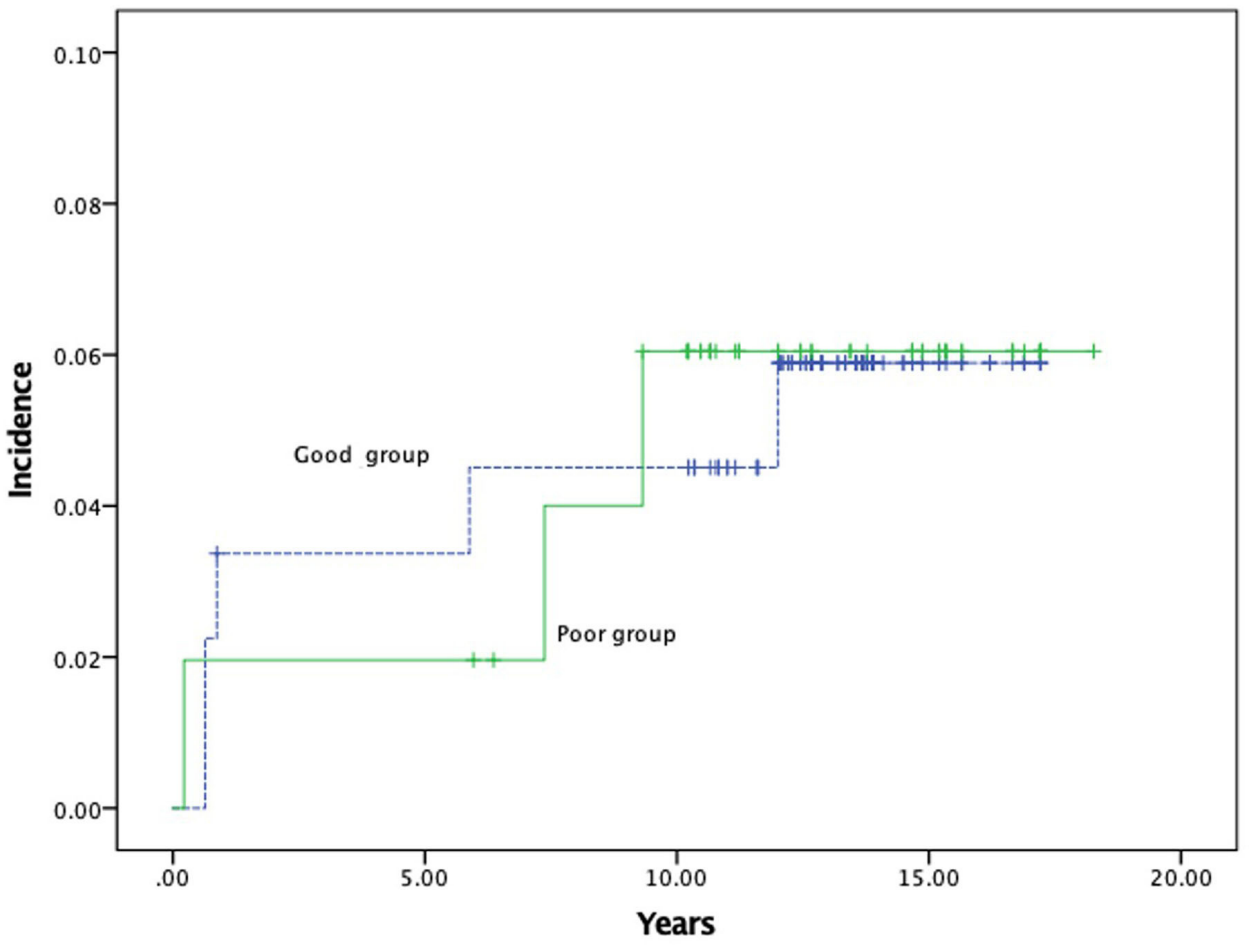
The Kaplan–Meier curves for stroke events between good and poor postoperative collateral circulation group during long-term follow-up.

A total of nine strokes (11.5%) occurred in 78 patients during follow-up. In total, four patients (5.1%) demonstrated ischemic stroke, while 5 (6.4%) showed hemorrhagic stroke. The annual rates of stroke were calculated to be 0.73% per person per year. Overall, the 1-, 5-, and 10-year actuarial stroke rates were 2.7 ± 1.3, 7.5 ± 2.4, and 12.3 ± 3.1%, respectively. In the good postoperative collateral circulation group, recurrent strokes were observed in five hemispheres (cerebral infarction in 2 and hemorrhage in 3). Furthermore, no hemisphere with postoperative Matsushima grade 0 suffered any recurrent strokes at long-term follow-up. In the poor postoperative collateral circulation group, recurrent strokes were observed in four hemispheres (cerebral infarction in 2 and hemorrhage in 2). No significant differences were found between these two groups (*p* = 0.917, Figure 4). In the progression group, recurrent strokes were observed in five hemispheres (cerebral infarction in 2 and hemorrhage in 3) among 26 hemispheres at long-term follow-up. In the stable group, recurrent strokes were observed in four hemispheres (cerebral infarction in 2 and hemorrhage in 2) among 130 hemispheres. The annual incidence rate of recurrent strokes was higher for the progression group than for the stable group.

Figures 2, 3 show two representative cases.

## Discussion

Indirect revascularization is an essential component of the surgical treatment of MMD. This procedure improves cerebral perfusion by attaching pedicled, vascularized grafts to the cortical surface and facilitating the ingrowth of neoangiogenesis (3, 4). There are a few pieces of literature focused on the progression of neoangiogenesis after indirect revascularization (10, 15). However, there are still many unknowns about this procedure. Does neovascularization continuously develop with time or with the progress of the internal carotid artery? When does neovascularization substantially and fully develop after surgery? Can the development of collateral circulation through indirect revascularization decline after an extended period of time? In this retrospective study, revascularization after indirect bypass surgery of 145 hemispheres in 78 patients with MMD on catheter angiography at short-term (7.4 months) follow-up and long-term (12.5 years) follow-up was compared, aiming to answer the questions listed above.

The current study compared both qualitative and quantitative measurements of neoangiogenesis after EDAS on lateral views of DSA. The findings showed the Matsushima grades of the same hemisphere were higher at long-term follow-up compared with that at short-term follow-up. The same trends were observed by quantitative measurements. These findings indicated that postsurgical neoangiogenesis continuously improves with time, which was consistent with other studies (9, 15). Most importantly, no attenuation was observed in any hemisphere during long-term follow-up. The long-term stability of postsurgical neoangiogenesis guaranteed the long-term effect of indirect revascularization. To our knowledge, the mean duration of follow-up in this study was the longest to date among published angiographic investigations. However, an article showed that the formation of collaterals was established within 6 months after surgery, and the effect of revascularization did not change drastically after 6 months. They believed that arterial neovascularization was approximately finalized within 6 months after indirect bypass surgery (10). The inconsistent result might be attributable to their relatively short-term follow-up (1 year). Therefore, even if the collateral grade at the time of follow-up is not as high as expected, it is likely that it will improve with time. Even if this study demonstrated that the Matsushima grades of the same hemisphere were higher at long-term follow-up compared with that at short-term follow-up, the growth processes of neoangiogenesis began to slow after 7.4 months. Six- or seven-months following surgery was a critical time window for patients with MMD after indirect bypass surgery, during which potential risks still exist due to unreliable revascularization, patients requiring special care, and the critical maintenance of cerebral blood flow. Potential pharmaceutical therapy might be given to facilitate the growth of new vessels during this time window if applicable.

A previous study suggested that surgical procedures would significantly accelerate the stage progression of the ipsilateral hemisphere in pediatric patient with MMD (16). This study showed that stage progression with different levels was observed in 50 operative hemispheres and only one non-operative hemisphere. As the relatively small sample size of the non-operative hemisphere, we could not draw a definitive conclusion. However, considering the more rapid progress of stage progression in short-term compared with that at long-term follow-up, there is a strong possibility that cerebral revascularization plays an important part in stage progression. The mechanism by which cerebral revascularization will affect the stage progression of the ipsilateral hemisphere is not clear. The most likely reason is that neo-formative collaterals originating from the extracranial artery provide new and better support to the ischemic brain, thus, decreasing the necessity of these weak branches and leading to their occlusion, as proposed by previous studies (10, 16, 17). Accordingly, this study showed that stage progression was significantly correlated with the postsurgical Matsushima grade at short-term follow-up after cerebral revascularization.

By contrast, the progression of Suzuki stages at long-term follow-up is likely attributed to the natural progression of the disease, as MMD is a progressive and occlusive cerebrovascular disease. The relation between the stage progression and postsurgical neoangiogenesis at long-term was different from that at short time after cerebral revascularization. With the natural progression of MMD, neo-formative collaterals originating from the extracranial artery could continuously develop. Two typical cases in this study demonstrated this process well. Interestingly, EDAS in these cases may be regarded as an unhelpful operation as no or little neo-formative collaterals originating from ECA were observed at the beginning. However, neoangiogenesis began to develop and became burly in long-term accompanied by the progression of the ICA. The results indicated that EDAS could play an important preventive role in preventing future ischemia, even if it did not appear to work at short-term follow-ups. Therefore, indirect revascularization should be suggested in young patients with even the slightest ischemia. In the absence of a comparison with direct revascularization in our present study, we could not conclude that indirect revascularization has an advantage in the long-term. A long-term follow-up angiographic study found that an indirect effect of formation of new vessels in the hypoperfused regions of the brain seemed to be prevalent after extracranial-intracranial arterial bypass. The result showed indirect revascularization demonstrated advantageous long-term effects.

This study showed that the 1-, 5-, and 10-year actuarial stroke rates were 2.7 ± 1.3, 7.5 ± 2.4, and 12.3 ± 3.1%, respectively. The result was slightly higher compared with our previous studies (6, 7, 12, 13). The discrepancy was mainly attributed to selection bias. In our study, some patients were admitted to the hospital and received long-term follow-up DSA due to recurrent infarction or hemorrhage. Although long-term follow-up was requested for all patients, a significant portion of patients refused due to their good recovery with no sign of ischemia or bleeding. We found that long-term clinical outcome was not related to the postsurgical Matsushima grade, indicating an inconformity between clinical outcome and radiological outcomes. Among the nine recurrent strokes, four were infarctions, including two occipital lobe infarctions, one frontal lobe infarction, and one contralateral parietal lobe infarction without revascularization surgery. This meant that no infarction was related to insufficient neoangiogenesis induced by indirect revascularization. This may also indicate a more positive effect of repeat bypass surgery for the frontal/occipital area in refractory MMD. Among the recurrent strokes, five were hemorrhages. Furthermore, three of five patients initially presented with ischemia. Furthermore, hemorrhagic onset was recognized as an independent predictor of poor neoangiogenesis after indirect bypass surgery. Whether indirect surgery can effectively prevent rebleeding remains a controversial issue. Further research focusing on reducing rebleeding rate after indirect revascularization might be needed.

This study had a few limitations. First, this study had a small number of patients; nonetheless, our findings can be considered significant because MMD itself is rare, and long-term follow-up is not easy in terms of clinical and angiographic states. Second, a selection bias may exist given the study's retrospective design. To limit this bias, all the consecutive patients underwent surgery by a single surgeon. Third, perfusion data were not included in this study owing to the use of different diagnostic methods, such as positron emission tomography, single-photon emission computed tomography, and computed tomography perfusion presurgery, postsurgery, and during long-term follow-up in our early cases. Fourth, the only procedure included in this study was EDAS. Other types of indirect surgery, including multiple burr holes, encephaloduromyoarteriosynangiosis (EDMAS), and encephalodurogaleosynangiosis (EDGS), were not investigated. Further research with a larger set of clinical data and including more surgical types might be needed to validate the progression of neovascularization development in MMD.

## Data Availability Statement

The datasets presented in this article are not readily available due to ethical and privacy restrictions. Requests to access the datasets should be directed to the corresponding authors.

## Ethics Statement

The studies involving human participants were reviewed and approved by the Research Ethics Board at the Fifth Medical Center of Chinese PLA General Hospital. Written informed consent to participate in this study was provided by the patients/participants or patients/participants' legal guardian/next of kin.

## Author Contributions

X-YB: drafting the article; Q-NW: acquisition of data, Statistical analysis; X-PW: acquisition of data, analysis and interpretation of data; R-MY and Z-XZ: acquisition of data; QZ and D-SL: technical support; LD: conception and design, critically revising the article. All authors contributed to the article and approved the submitted version.

## Funding

This study was supported by grant from the National Natural Science Foundation of China (Grant No. 82171280).

## Conflict of Interest

The authors declare that the research was conducted in the absence of any commercial or financial relationships that could be construed as a potential conflict of interest.

## Publisher's Note

All claims expressed in this article are solely those of the authors and do not necessarily represent those of their affiliated organizations, or those of the publisher, the editors and the reviewers. Any product that may be evaluated in this article, or claim that may be made by its manufacturer, is not guaranteed or endorsed by the publisher.
